# How Italian Middle School Adolescents Conceptualize and Navigate Cyberbullying: A Qualitative Analysis of Definitions, Behaviors, Roles, and Coping Strategies

**DOI:** 10.3390/bs16030435

**Published:** 2026-03-17

**Authors:** Laura Menabò, Felicia Roga, Silvia Fernández Gea, Debora Ginocchio, Annalisa Guarini

**Affiliations:** 1 Department of Psychology “Renzo Canestrari”, University of Bologna, 40127 Bologna, Italy; felicia.roga@unibo.it (F.R.); annalisa.guarini@unibo.it (A.G.); 2 Department of Psychology, Faculty of Psychology, University of Almeria, 04120 Almería, Spain; sfg023@ual.es; 3 Faculty of Law, University of Modena and Reggio Emilia, 41121 Modena, Italy; debora.ginocchio@unipr.it

**Keywords:** cyberbullying, focus group, cyberbullying definition, cyberbullying behaviors, cyberbullying roles, cyberbullying coping strategies, qualitative

## Abstract

Backgrounds: Cyberbullying represents a major concern for students, yet most studies rely on quantitative and adult-centered perspectives. Understanding adolescents’ views on cyberbullying is crucial for prevention. Method: We conducted sixteen focus groups with 220 Italian middle school students (ages 11–13). Transcripts were inductively analyzed to identify domains, core ideas, and the occurrence of categories (general, typical, variant) using the Consensual Qualitative Research method. Results: Four main domains emerged: definitions, behaviors, roles, and coping strategies. Adolescents defined cyberbullying as a hostile online interaction marked by publicity, often followed by anonymity; few mentioned repetition. Direct acts such as insults, threats, and non-consensual image sharing were viewed as the most harmful behaviors, followed by impersonation and identity theft, while online challenges and other forms were less mentioned. Students mainly perceived cyberbullying as a dyadic interaction between bully and victim, showing limited awareness of pro-bullies, few references to bystanders, and no mention of defenders. Finally, participants focused on victims’ responses with little attention to bystanders’ coping strategies. Conclusions: By revealing a nuanced understanding of cyberbullying, adolescents emphasize the need for prevention programs that not only address online risks but also build on their own language, perspectives, and experiences.

## 1. Introduction

Cyberbullying is a widespread and evolving form of peer aggression among adolescents, associated with significant psychological and social consequences, including anxiety, depression and suicidal behaviors (Coelho et al., 2022; Sarhangi et al., 2023). In Italy, it represents a particularly pressing concern among youth aged 11 to 15 years (Istituto Superiore di Sanità, 2022). At the same time, as digital technologies and online interactional norms rapidly change, the meanings, expressions, and social dynamics of cyberbullying continue to transform (Scheithauer et al., 2021).

Despite extensive research, much of the literature relies on quantitative approaches and predefined constructs (Slonje et al., 2025; Warkentin et al., 2026). While these studies have clarified prevalence rates and correlates, they provide limited insight into how adolescents themselves define, interpret, and evaluate cyberbullying within their lived digital experiences. Consequently, young people’s subjective perspectives remain comparatively underexplored.

This gap is particularly relevant in the Italian context, where recent qualitative investigations on adolescents’ experiences of cyberbullying are still scarce. Therefore, a deeper understanding of how youth conceptualize cyberbullying is essential not only for theoretical refinement but also for developing prevention and intervention strategies that resonate with their everyday realities.

To address this gap, the present study adopts a qualitative approach to explore four interconnected domains: (1) adolescents’ definitions of cyberbullying, (2) the identified behaviors, (3) their understanding of roles within cyberbullying dynamics, and (4) the coping strategies they recognize as victims and bystanders.

Indeed, while several qualitative studies (e.g., Mischel & Kitsantas, 2023; Dahal, 2023; Erbiçer et al., 2022) have explored specific aspects of online aggression, few have provided a comprehensive understanding of how adolescents themselves integrate definitions, roles, behaviors, and coping strategies into a coherent representation of cyberbullying.

By addressing these interrelated domains, this study aimed to offer a socially grounded account of adolescents’ experiences with cyberbullying. Centering their voices provided critical insights into how young people understand and manage online aggression, and contributed to developing prevention and intervention programs that align with both their digital realities and the rapidly changing nature of cyberbullying.

### 1.1. Cyberbullying Definition

Cyberbullying was initially conceptualized as an intentional and repeated form of aggression enacted through electronic means, reflecting the core criteria of traditional bullying while situating them in digital contexts (Smith et al., 2008). Notably, nearly two decades later, a recent international consensus study involving 149 researchers from 36 countries still defined cyberbullying in similarly broad terms as bullying perpetrated through electronic devices within social networks, by both known and unknown individuals (Zych & Farrington, 2026).

Despite this apparent continuity, conceptual clarity remains limited. A systematic review (Zhang et al., 2022) found that many empirical studies adopt inconsistent or unclear definitions, and institutional reports have acknowledged the absence of a universally agreed framework (European Commission, 2024). In addition, while researchers debate conceptual criteria, students themselves may interpret cyberbullying in very different ways.

Qualitative studies indicate that adolescents prioritize intention to harm and the victim’s suffering when defining cyberbullying, while interpreting repetition and power imbalance more flexibly (Vandebosch & Van Cleemput, 2008; Naruskov et al., 2012). More recent evidence further supports this view, showing that even a single act may be considered cyberbullying if it reaches a wide audience (Mischel & Kitsantas, 2023). Indeed, digital-specific features, such as anonymity, public visibility, and the permanence of online content, are perceived as intensifying harm and shaping judgments of severity (Midamba & Moreno, 2018; Biernesser et al., 2023; Chun et al., 2024). Overall, adolescents’ definitions appear grounded less in abstract criteria and more in contextual, relational, and experiential factors.

### 1.2. Cyberbullying Behavior

Cyberbullying manifests in a wide range of behaviors that evolve alongside technological advancements, making its classification complex and, at times, contested (Scheithauer et al., 2021). A widely cited framework distinguishes between direct behaviors (e.g., insults or threats) and indirect behaviors (e.g., rumor spreading, exclusion, or harmful image sharing) (Willard, 2007; Doane et al., 2014; Iqbal & Jami, 2022). Other forms include impersonation, cyberstalking, and shaming practices, often targeting reputations and social norms (Keys & Bhogal, 2018; Sripa & Ninphet, 2024; Sreelatha, 2023).

Across qualitative studies, students reported a broad spectrum of behaviors, ranging from verbal attacks and threats to exclusion, identity misuse, and the non-consensual sharing of personal or intimate content (Jacobs et al., 2015; Midamba & Moreno, 2018; Mischel & Kitsantas, 2023). Importantly, these behaviors are not perceived as equivalent: adolescents frequently differentiate between forms of cyberbullying based on their perceived severity and the extent of harm inflicted on the victim (Mischel & Kitsantas, 2023).

Denigration and outing/trickery are often identified as especially harmful due to their intent to publicly damage the victim’s reputation (Iqbal & Jami, 2022; Mischel & Kitsantas, 2023).

Visual and sexual forms of cyberbullying, including the circulation of images, memes, or videos, are consistently described as among the most harmful experiences due to their public visibility and potential for rapid, uncontrollable dissemination (Midamba & Moreno, 2018; Chun et al., 2024). Qualitative studies also highlight the role of anonymity and limited adult supervision in facilitating behaviors such as password theft, extortion, and harassment via fake accounts, which are perceived as particularly threatening and difficult to stop (Biernesser et al., 2023; Martin et al., 2023). Taken together, these evidences underscore the importance of examining cyberbullying behaviors not only in terms of their form, but also in relation to how adolescents evaluate their harmfulness and social consequences.

### 1.3. Cyberbullying Roles

Cyberbullying is a complex social phenomenon embedded in broader interpersonal and group dynamics (Mishna et al., 2022). Beyond the bully–victim dyad, roles such as pro-bullies, defenders, and passive bystanders also emerge online (Pyżalski et al., 2022). Pro-bullies may reinforce aggression (e.g., by liking or sharing harmful content), defenders may support victims or report incidents, whereas passive bystanders often remain inactive, potentially legitimizing the aggression (Bastiaensens et al., 2015; Pozzoli & Gini, 2020; Pyżalski et al., 2022).

At the qualitative level, cyberbullies are often described as acting anonymously, impulsively, or for entertainment, sometimes framing aggression as a joke, and avoiding face-to-face confrontation (Mishna et al., 2022; Pyżalski et al., 2022). Such behaviors have also been linked to factors such as peer approval, low self-esteem, exposure to violence, or family difficulties (Machado et al., 2024). Cybervictims, in turn, are portrayed as experiencing emotional distress and powerlessness, particularly when aggression is anonymous or publicly visible (Mischel & Kitsantas, 2023; Pyżalski et al., 2022). Cybervictimization often targets perceived vulnerabilities, physical, psychological, or sociodemographic, and may be intensified by relational proximity to the aggressor (Pyżalski et al., 2022; Machado et al., 2024). Adolescents associate cyberbullying with serious consequences, including low self-esteem, social isolation, and, in some cases, suicidal ideation or disciplinary sanctions (Biernesser et al., 2023).

Concerning the other roles, bystanders emerged as a distinct group, with varying degrees of involvement. The study by Iqbal and Jami (2022) found that many people hesitate to intervene due to fear of ridicule or personal risk, although some would act in support of friends or family. Machado et al. (2024) described a broad spectrum of bystander behaviors, including supporting the victim, stopping the spread of content, reporting incidents, talking to the aggressor, or blocking them online. Notably, across qualitative studies, bullies and victims are described in greater depth than bystanders, reflecting adolescents’ tendency to frame cyberbullying primarily as a dyadic interaction, with less emphasis on the broader social context.

### 1.4. Coping Strategies in Cyberbullying

Coping refers to the strategies individuals use to manage stressful or threatening experiences (Aldwin, 2009), and in the context of cyberbullying, it plays a crucial role in moderating its psychological impact (Spears & Taddeo, 2021). Applied to cyberbullying, coping responses are shaped by the nature of the aggression, developmental factors, and the affordances of digital environments, and include emotional, social, and technical strategies, such as blocking, confrontation, avoidance or disengagement, and seeking social support (Spears & Taddeo, 2021; Raskauskas & Huynh, 2015; Perren et al., 2012).

Qualitative studies show that adolescents most often rely on technical and avoidant strategies to regain a sense of control, such as blocking users, remaining silent, disconnecting, or deleting content (Jacobs et al., 2015; Dahal, 2023; Midamba & Moreno, 2018). Inaction is often described as a deliberate choice aimed at preventing escalation, avoiding retaliation, or preserving social relationships (Dahal, 2023; Erbiçer et al., 2022; Jacobs et al., 2015). Fear of social or familial consequences may further inhibit disclosure, particularly in cases involving non-consensual sharing of intimate material (Martin et al., 2023). Nonetheless, some adolescents seek support from trusted others, like family or friends, recognizing the protective role of emotional validation and practical help (Dahal, 2023; Erbiçer et al., 2022; Jacobs et al., 2015). A smaller group adopts confrontational or self-affirming responses, directly addressing the aggressor or displaying confidence and indifference to reassert agency and protect self-esteem (Vandebosch & Van Cleemput, 2008; Jacobs et al., 2015; Dahal, 2023). Despite extensive literature on victims’ coping strategies, far less attention has been paid to how bystanders cope with cyberbullying. Quantitative evidence suggests that bystanders most often choose non-intervention, and, when they do, they offer offline support to victims or seek help from adults (Zhao et al., 2023), but adolescents’ own evaluations of these responses and their perceived effectiveness remain largely unexplored.

### 1.5. Research Questions

The present study adopts an integrative qualitative approach to explore adolescents’ narratives about cyberbullying. The study addresses the following research questions:

(1) How do adolescents define and conceptualize cyberbullying?
(2) Which behaviors do adolescents identify as cyberbullying?
(3) How do adolescents perceive and describe the roles involved in cyberbullying situations?
(4) Which coping strategies do adolescents report from the perspectives of victims and bystanders?

Given the exploratory nature of the study, the findings are expected to highlight aspects that are consistent with existing literature while also offering new insights, particularly regarding possible variations in how adolescents interpret and respond to cyberbullying, which may open new directions for reflection and future research.

## 2. Materials and Methods

### 2.1. Participants

The study involved 16 focus groups conducted in seven lower secondary schools across the Emilia-Romagna region in Italy during 2022.

Each focus group represented a single class from either the sixth or seventh grade. A total of 220 students participated in the research, comprising 114 boys (52%) and 106 girls (48%). Approximately 9% of the participants (20 students) had a foreign background. The average age of the sample was 11.3 years (*SD* = 0.6).

### 2.2. Procedure

School principals were first contacted via email and provided authorization for their schools to participate in the study. Subsequently, informed consent was obtained from students’ families through the school staff, allowing students to participate in the study, the analysis of the collected data, and the anonymous publication of the findings. The focus group discussions were guided using a semi-structured protocol designed to encourage open conversations around the aforementioned topics related to cyberbullying. The questions were intentionally straightforward to allow students to express their views freely. Prompts included:

1. How would you define cyberbullying?
2. Who are the people typically involved in cyberbullying?
3. What strategies do you think can be used to address this issue?

Notably, the first question was used to explore both students’ definitions of cyberbullying and their descriptions of the specific behaviors associated with it.

All sessions were facilitated by at least one member of the research team. Discussions were audio-recorded and later transcribed word-for-word. The qualitative analysis followed the Consensual Qualitative Research (CQR) method (Hill, 2012), ensuring rigor and systematicity throughout the process. The CQR approach continues to be widely applied in recent qualitative research (e.g., Frawley et al., 2024; Shah et al., 2024; Horanicova et al., 2024), supporting its ongoing methodological relevance.

The research team consisted of four members: three core analysts with varying expertise in psychology and bullying research (including a trainee psychologist, a research psychologist, and a psychotherapist), and an external auditor with extensive academic experience in the field.

To address potential power dynamics and ensure equal participation, early meetings focused on fostering a collaborative environment where each member’s input was equally valued. The first author, after a thorough study of CQR methodology, developed a coding system in Italian to promote inclusivity for all team members. The team then engaged in two preparatory sessions to practice the approach.

### 2.3. Domain Construction and Coding Process

In line with Hill’s (2012) recommendations, the team generated coding domains inductively rather than using a pre-defined list. Each core team member independently segmented the transcripts of three focus groups and proposed domain structures. These initial versions were applied and refined through the analysis of additional transcripts, with the process iterated until data saturation was reached and no new domains emerged.

After finalizing the list of domains, the first author coded the remaining transcripts, which were subsequently reviewed by the team. The auditor (the last author) then examined the finalized domain framework. Each unit of relevant text was assigned to one or more domains, and any ambiguous data were temporarily grouped under a provisional “Other” category pending further discussion.

### 2.4. Core Ideas

Core ideas represent concise summaries of participants’ statements that preserve the original meaning while enhancing clarity and comparability. Team members paraphrased students’ expressions, without adding interpretations or assumptions, to distill the essential message from each response. Once familiar with the approach, the team adopted a rotating system: one member drafted the core ideas while the others reviewed and refined them. Redundancies and irrelevant content were removed, and pronoun use was standardized to ensure consistency. The final version of each set of core ideas was validated by the external auditor.

### 2.5. Cross-Analysis

Following the auditor’s review, all core ideas were consolidated into a master document, maintaining references to the original transcript’s case and turn numbers. The team collaboratively analyzed the data within each domain to identify common patterns and construct thematic categories.

This categorization process was iterative. As themes emerged, categories were revised until a stable and comprehensive structure was reached. Each category was then classified as:

- General: identified in 14–16 focus groups (over 75%).
- Typical: found in 5–13 focus groups (26–75%).
- Variant: mentioned in 1–4 focus groups (up to 25%).

This classification enabled a clear understanding of the consistency and relevance of different perspectives on cyberbullying across the sample.

The original language of the focus group transcripts was Italian; therefore, the student quotes below are English translations made by the first author to facilitate the reader’s understanding.

## 3. Results

### 3.1. Domain: Definition of Cyberbullying

The first domain explored how students define cyberbullying. A general core idea highlighted that cyberbullying is seen as aggressive and offensive behavior occurring online. Another general idea concerned the uncontrolled spread of harmful content, which students described as rapid and difficult to stop, increasing its impact.

As a typical core idea, students emphasized the role of anonymity, which can encourage more hostile behavior.

Finally, a variant core idea challenges the notion that repetition is necessary: some students pointed out that even a single act of cyberbullying can cause significant harm (Table 1).

#### 3.1.1. General

- **Category: Hostile Online Communication**. At the general level, students defined cyberbullying as aggressive and offensive behaviors that occur online:

*“…I think it’s always a form of offense that doesn’t involve physical harm, only verbal offense, and that happens online.”*

(Focus group 15)

*“If I keep saying bad things to you for a whole day, like “go kill yourself,” then yes, that counts as cyberbullying.”*

(Focus group 2)

- **Category: Uncontrolled Spread**. It is highlighted that once harmful content is posted online, it can spread quickly and uncontrollably, reaching a wide audience.

*“I post a picture on social media, a cyberbully takes it, edits it, and republishes it. Then maybe another cyberbully sees it, sends it to a friend, who then shares it with another friend. To me, it’s a chain, a vicious circle that never ends. So, it’s not enough to simply delete that picture from my profile.”*

(Focus group 6)

*“In my opinion, the worst thing about cyberbullying is that if they make an embarrassing video of you, they send it to everyone.”*

(Focus group 8)

*“They posted embarrassing photos of him, I won’t say why, and then the whole school group who saw them started laughing at him when he walked down the hallways.”*

(Focus group 3)

#### 3.1.2. Typical

- **Category: Anonymity and Concealment.** Students emphasized that cyberbullying often occurs behind the screen and may involve unknown individuals. The anonymity provided by technology can facilitate aggressive behavior and disinhibit the cyberbully.

*“When someone is insulted through a screen without even knowing the person on the other side.”*

(Focus group 1)

#### 3.1.3. Variant

- **Category: No Need for Repetition**. Some students pointed out that cyberbullying can have a strong emotional impact even when it occurs only once, suggesting that repetition is not necessary for harm to be inflicted.

*“Um…when I was bullied on the phone, it really bothered me, even if it happened only once.”*

(Focus group 4)

### 3.2. Domain: Cyberbullying Behaviors

This domain focused on the different forms of behaviors that adolescents associate with cyberbullying. At the general level, students described a wide range of actions that can occur across various online platforms, from social media to gaming environments. Cyberbullying was mainly characterized by hostile or threatening messages, insults, and the sharing of embarrassing or nonconsensual content. Typical ideas included forms of identity misuse, such as impersonation or the creation of fake profiles, which allow perpetrators to act anonymously. Variant ideas referred to extreme and dangerous behaviors, such as online challenges with potentially harmful outcomes, and to distinctions students made between less and more severe forms of cyberbullying depending on their context and intent (Table 1).

#### 3.2.1. General

- **Category: Variety of Platforms**. Students reported that aggressive and abusive cyberbullying behaviors occur online on different digital platforms, including social media, mainly Instagram, TikTok, WhatsApp, and Facebook, or online game platforms.

*“Cyberbullying is the one that they bully you online […] through the cell phone, for example, if you post pictures on Instagram.”*

(Focus group 11)

*“(Cyberbullying) is a situation that could happen on social media […] TikTok, Instagram…”*

(Focus group 6)

*“The bully teases a person but through social media, internet, TikTok, […] especially WhatsApp.”*

(Focus group 13)

- **Category: Unpleasant Comments and Insults**. Many cyberbullying behaviors involve posting nasty, offensive, or insulting comments on other people’s content. Sometimes these comments were about people who are perceived to be inferior because of some trait, physical or character, which is mocked.

*“I used to see that in the comments (people) always wrote insults to individuals who either posted videos or who commented […] even if you don’t know them, they can offend you.”*

(Focus group 1)

*“She was being bullied on social, on TikTok, for the videos she was making…because she was showing her face, so she was being bullied, she was being told like “you have crooked, beaver teeth.”*

(Focus group 5)

*“(Cyberbullying) may be teasing like […] you public a photo, and they criticize it.”*

(Focus group 16)

- **Category: Threats.** Cyberbullying was also often associated with threats from somone who wants to intimidate or induce you to do something. This can have a significant impact on victims, including feeling fear or anxiety about what might happen if one is not compliant with the cyberbully.

*“In cyberbullying, you can threaten others.”*

(Focus group 12)

*“…they were telling me do this, go that way, […] and I didn’t want to do all these things […] and then, at the end, I remember they threatened me with death like, “If I catch you, I’ll strangle you”. And I came out of the online room terrified, I was afraid.”*

(Focus group 2)

- **Category: Sharing Embarrassing Content.** Some students described cyberbullying as the nonconsensual posting of embarrassing or modified personal information, photos, or videos on online platforms. This behavior can cause damage to the victims’ reputation.

*“Cyberbullying is like a mockery towards other people, like having ugly pictures sent around.”*

(Focus group 1)

*“In my opinion, (cyberbullying) is also a person who secretly takes pictures of you and then puts them on the web without your consent.”*

(Focus group 10)

*“They took a picture of me and edited it, I had cat ears […] basically, do you know the stickers on WhatsApp? They created one for me with a face […] with an ugly face.”*

(Focus group 9)

#### 3.2.2. Typical

- **Category: ID theft/impersonation.** Some students mentioned online identity theft or impersonation as another frequent cyberbullying behavior. In this case, a person may log into someone else’s social account and, pretending to be this person, share content or write to people inappropriately.

*“Maybe someone could steal your account, pretend to be you, put up embarrassing pictures of you, write to some of your friends, offend them…or maybe write to a random person.”*

(Focus grop 13)

*“My mother’s Facebook account was stolen from her, and I learned that they had stolen it through a family chat, and they had posted something about my aunt that was not appropriate.”*

(Focus group 7)

*“(The cyberbully) may be someone who hides under someone else’s identity… he pretends to be him, but he is another person.”*

(Focus group 14)

- **Category: Fake Identity.** Online interactions where the distance between people was provided by a screen can give rise to another behavior, often mentioned by students, namely, the creation of fake profiles. A person can register on an online platform using a fake identity, entering data that is not their own, so that they can write inappropriate content and insult others without fear of being discovered and identified as the perpetrator.

*“It’s cyberbullying when someone insults you on social media, and you don’t even know who it is, because they might be using a fake identity.”*

(Focus group 3)

*“To me, cyberbullying is when someone who doesn’t dare to confront others face-to-face hides behind a screen and insults people from fake profiles.”*

(Focus group 3)

#### 3.2.3. Variant

- **Category: Dangerous Challenges.** In some cases, cyberbullying was associated with challenges that can be physically dangerous or life-threatening, such as the case of the “Blue Whale”. These extreme behaviors can have serious consequences for the mental and physical health of victims.

*“There are people behind the screen who offend […] but (cyberbullying) can maybe even be threats or even challenges that could cause you to die.”*

(Focus group 11)

*“For example, in the games they offer you challenges and then say that if you don’t do them well, they will kill your parents […] I remember on the news they said that a girl had strangled herself.”*

(Focus group 7)

- **Category: Severity Differences**. Students distinguished between less severe cyberbullying, like bullying in online games, and more serious forms that specifically target individuals, often based on personal aspects, like physical appearance. This distinction reflected the variety of behaviors included in the term “cyberbullying”.

*“I think that the consequences are less serious in online games than on social media.”*

(Focus group 4)

### 3.3. Domain: The Roles Involved

The third domain concerned the roles involved in cyberbullying incidents. Across most focus groups, students focused primarily on the cyberbully and the cybervictim, offering detailed narratives of their perceived psychological traits. A smaller number of groups mentioned pro-bullies, those who support or reinforce the bully’s actions, while only a few referred to bystanders, often highlighting their lack of intervention. Interestingly, the defender role was never explicitly mentioned by participants (Table 1).

#### 3.3.1. General

- **Category: Cyberbully’s profile**. Students tended to describe the cyberbully as cowardly and with no friends who engage in cyberbullying to avoid detection and face real consequences.

*“Those who bully others online are cowards who don’t want to show their faces, so they make fun of people instead…”*

(Focus group 16)

*“…those that scare people through social media, offend them like that, they usually don’t even have a lot of friends because they’re usually kind of closed … so they lock themselves inside the room and start offending…”*

(Focus group 2)

The words of some students revealed that some people may resort to cyberbullying as a means of venting their frustrations.

*“I think cyberbullying is sometimes people venting about a bad day by going on social media to criticize others.”*

(Focus group 9)

In other cases, cyberbullying is related to personal quarrels or personal problems of the bullies, as is the case with bullying.

*“…I don’t think people who cyberbully, they just do it because they woke up bad, maybe there are also problems behind it, anyway, exactly like in bullying…”*

(Focus group 1)

- **Category: Cyber-victim’s profile.** Victims of cyberbullying were often described as being weaker, sensitive, or showing weaknesses. This shows how bullies deliberately choose individuals they perceive as vulnerable.

*“…like if somebody is sturdier or has a little bit more trouble about something… [the cyberbully] teases him.”*

(Focus group 10)

*“In my opinion, the most fragile people and those who cannot defend themselves are especially targeted.”*

(Focus group 3)

Students pointed out that on social media, people who are less popular, i.e., who have fewer followers, can be targeted.

*“[…] are targeted […] those who have characteristics […] like… if someone has few followers, they get picked on.”*

(Focus group 11)

It is also emphasized how cyberbullying can have strong consequences for victims, even including suicide.

*“…there are some people who from the stress also go to suicide…”*

(Focus group 4)

*“… I know of people who, because of that fact (cyberbullying) have really…depressed.”*

(Focus group 13)

#### 3.3.2. Typical

- **Category: Pro-bullies**. Students suggested that in cyberbullying incidents, there are also people who forward the offensive content initially disclosed by the cyberbully. In this way, they become cyberbullies’ indirect accomplices.

*“…usually the bully has supporters who reinforce his message…”*

(Focus group 1)

In some cases, these may be people supporting the bully to prevent them from picking on themselves.

*“They might be people who also flank the bully, who have joined him so that they won’t be teased by him.”*

(Focus group 11)

#### 3.3.3. Variant

- **Category: Passive Bystanders.** Students complain of a general lack of online victim advocacy, indicating that other users do not intervene to protect them.

*“Other people see what’s happening online, but they don’t do anything, they prefer not to get involved.”*

(Focus group 13)

### 3.4. Domain: Coping Strategies

Coping strategies for cyberbullying can be divided between strategies adopted by bystanders and victim strategies (Table 1).

#### 3.4.1. General

- **Category: Victim Strategies**. A common approach was to delete or hide offensive content. Students mention removing embarrassing photos or inappropriate comments from their social profiles. This strategy was often used to protect one’s online reputation.

*“If an embarrassing picture of me went around, I would delete it from my profile; if it wasn’t on my profile, I couldn’t do anything about it…”*

(Focus group 3)

Some students suggested responding defensively to the insults, trying to defend themselves without worsening the situation.

*“If I were a victim of cyberbullying, I would physically attack the cyberbully.”*

(Focus group 9)

Blocking the cyberbully was another common strategy. This prevents the cyberbully from continuing to communicate with the victim or see their online content.

*“You can block the cyberbully, and even if he keeps posting things at least you don’t see them anymore.”*

(Focus group 14)

Many students suggested sharing the experience with a trusted adult, such as parents or teachers, or with friends. This can offer emotional support and help find solutions to the cyberbullying situation.

*“If my picture were posted without my consent, I would talk to my parents or another adult I trust… maybe I’d ask my friends to help me talk about it with them.”*

(Focus group 10)

However, not everyone agreed on reporting what happened to their parents, as there is a risk of having their social media taken away.

*“Reporting to parents can result in them stopping you from using social media and you can no longer hear from your friends either.”*

(Focus group 1)

Other participants suggested immediately reporting the offensive account or content to online platforms or relevant authorities. This can help remove harmful content and identify the cyberbully.

*“I would report the account immediately without even responding.”*

(Focus group 7)

*“When you are a victim of cyberbullying, it can be helpful to call the postal police to track down the attackers.”*

(Focus group 1)

Some students recommend taking a break from social media if they are experiencing cyberbullying. This allows them to avoid further emotional harm and focus on positive interactions in real life.

*“I think that if you are being cyberbullied, you can take a break from your phone, devoting yourself to real friends and experiencing the world beyond the Internet.”*

(Focus group 16)

Ignoring negative comments was another mentioned strategy. Some students believed that not responding to online provocations is the best response to avoid fueling the cyberbully’s behavior.

*“Dealing with nasty comments on social media like Instagram by ignoring, not responding, and continuing to use social media; putting on a show of crying and feeling sorry for yourself serves no purpose.”*

(Focus group 4)

Some students also mentioned the possibility of doing nothing

*“It’s not something real, so I wouldn’t do anything.”*

(Focus group 9)

*“If they don’t insult me, I don’t care, if they insult I still don’t care… because then they continue and if they are insulting you then maybe they insult you more and more.”*

(Focus group 12)

#### 3.4.2. Typical

- **Category: Bystander Strategies.** Defending the victim can be difficult. Students said they would only intervene if they were friends with the victim (without specifying how), while others explicitly stated that they would not intervene because they do not know who is on the other side. It is also mentioned that defending online is not easy because even if you decide to write comments against the cyberbully, they can be deleted with a click.

*“I mean…Defending the victim in the virtual context is very difficult, even if this is a friend of yours, everyone else you don’t know.”*

(Focus group 7)

*“To defend the victim you might write messages against the bully, but he deletes them with a click.”*

(Focus group 3)

*“If I were the victim, I would talk to someone about it; if I were a witness, I’d try to support the victim.”*

(Focus group 14)

## 4. Discussion

The present study aimed to explore how adolescents define and interpret cyberbullying, the behaviors they recognize as cyberbullying, the roles they attribute to those involved and the strategies they adopt to cope with it. In the following sections, the discussion is organized around the four domains that emerged from the analysis: definition, behaviors, roles and coping strategies.

### 4.1. Definition

Within the first domain, the definition of cyberbullying, two elements emerged as particularly central in adolescents’ perspectives. First, cyberbullying was consistently described as a form of aggression, manifested mainly through offensive or hostile online communication. This finding confirms qualitative studies (e.g., Biernesser et al., 2023; Naruskov et al., 2012) and underscores aggression as the most recognizable core of the phenomenon. Second, participants strongly emphasized the uncontrolled spread of harmful content. This perception aligns with more recent work identifying publicity and virality as crucial components of digital victimization (Biernesser et al., 2023; Fernández-Antelo & Cuadrado-Gordillo, 2018), but also represents a novel contribution in that students themselves considered this aspect fundamental to their definition of cyberbullying.

At the typical level, adolescents frequently referred to anonymity as an element that exacerbates harm, consistent with prior studies (e.g., Biernesser et al., 2023). However, while considered important, anonymity appeared less central than uncontrolled spread. This interpretation resonates with Fernández-Antelo and Cuadrado-Gordillo’s (2018) quantitative findings, which showed that although Spanish adolescents recognized anonymity as a facilitator of online aggression, they did not regard it as a primary criterion defining cyberbullying.

At the variant level, some students argued that a single act can be enough to constitute cyberbullying. For these adolescents, the intensity of harm, particularly when linked to public humiliation, outweighed the importance of frequency. Although expressed by a minority, this perspective resonates with ongoing scientific debates (Menin et al., 2021) that question whether repetition should remain a necessary criterion. It is also noteworthy that repetition never emerged spontaneously in students’ own definitions. Taken together, these findings illustrate how adolescents differentiate between more and less central features of cyberbullying. Interestingly, the notions of intentionality and power imbalance, traditionally considered core definitional elements, did not emerge explicitly in students’ narratives. It is plausible that adolescents perceive these elements as implicitly embedded within other dimensions, such as publicity, which amplifies power differentials through audience exposure, or anonymity, which fosters a sense of impunity and asymmetry between aggressor and victim. This interpretation suggests that, in digital contexts, power and intent may be experienced more through structural features of online communication than through individual motives or visible dominance.

### 4.2. Behaviors

Our findings both confirm and expand current knowledge on cyberbullying behaviors. At a general level, students most frequently mentioned comments and insults, threats, and the sharing of embarrassing content as the most salient forms of cyberbullying (e.g., Feng, 2021; Iqbal & Jami, 2022; Jacobs et al., 2015). Interestingly, however, students in our study did not mention exclusion as a typical cyberbullying behavior, despite its frequent recognition in prior research. This omission may suggest that adolescents perceive exclusion as a less visible or less prototypical form of online aggression compared to direct attacks such as insults or threats, highlighting the need to further investigate how subtle relational behaviors are understood in digital contexts.

Our findings underscore the particular salience of non-consensual content sharing as a highly harmful form of cyberbullying. Adolescents portrayed image-based abuse as especially distressing, emphasizing its capacity to amplify exposure, shame, and loss of control due to its permanence and rapid dissemination. These perceptions reinforce the view that visual forms of aggression carry a distinct and intensified emotional impact in digital contexts (Martin et al., 2023; Chun et al., 2024)

At the typical level, impersonation and identity theft emerged as less frequently mentioned, despite being well documented in the literature (e.g., Marinoni et al., 2024; Sripa & Ninphet, 2024). This may reflect not only a perceptual differentiation, where adolescents distinguish between aggressive behaviors that directly target the victim’s self-image and those that manipulate their identity indirectly, but also the relative rarity of these incidents in their everyday experiences. In other words, impersonation and identity theft might be less salient to students simply because they occur less frequently within their peer contexts. Notably, the emotional distress caused by impersonation was reported as especially acute when such acts involved interactions with peers or the sending of inappropriate messages in the victim’s name, as these situations directly threaten reputation and social standing.

At the variant level, students distinguished different degrees of severity, considering insults during online gaming as less serious compared to attacks targeting physical appearance or personal identity, particularly when these occurred on platforms like WhatsApp or Instagram. It also suggests that adolescents engage in nuanced contextual evaluations of digital harm: while insults in gaming contexts may be normalized as part of the competitive environment, attacks that are persistent, personal, and shared in more intimate or socially meaningful spaces are experienced as significantly more damaging.

Taken together, these findings emphasize that while adolescents readily recognize direct and visible behaviors such as insults, threats, and humiliating content sharing, they tend to underreport or reframe more subtle or indirect forms, such as exclusion and impersonation. This indicates that their conceptualization of cyberbullying prioritizes overt, emotionally salient behaviors and contexts where peer reputation and identity are most at stake.

### 4.3. Roles

In the introduction, we noted that while research acknowledges multiple peer roles, students often frame cyberbullying primarily as a dyadic conflict. Our findings confirm and extend this pattern. With regard to cyberbullies, students described them as socially isolated figures, lacking the courage for direct confrontation, and therefore using the online environment as an extension of offline dynamics. However, our participants enriched this perspective by emphasizing that cyberbullying behaviors may be driven by multiple motives: not only frustration and the need to vent, but also the desire to entertain peers or the intention to follow others’ example. Importantly, students highlighted that cyberbullies are not always anonymous strangers; they may also be friends or acquaintances who disguise aggression as a “joke” or a harmless behavior. This perception underscores the ambivalence of online relationships, in which betrayal by familiar peers can amplify the emotional impact of victimization. It also suggests that adolescents’ sense of trust and belonging may be destabilized when aggression comes from known peers, an aspect that has received limited attention in prior research.

The figure of the cybervictim was consistently described as that of someone perceived as emotionally or socially vulnerable. Beyond individual fragility, students also pointed to digital status indicators, such as having few followers or limited online visibility, as risk factors for becoming a target. This finding highlights the growing role of online popularity and digital social capital in shaping peer hierarchies and vulnerability to aggression. Moreover, adolescents explicitly recognized the severe consequences of cybervictimization, mentioning shame, depression, and even suicidal ideation, demonstrating adolescents’ awareness of the profound emotional impact of the phenomenon.

Another key finding concerns the strong emphasis students placed on the main actors, bully and victim, while providing few references to other roles. This tendency to describe cyberbullying as essentially a dyadic conflict has already been noted in prior research (e.g., Menabò et al., 2025) and may be understood as both a cognitive and cultural narrative simplification. Adolescents may be less likely than adults to notice or articulate secondary roles due to more limited cognitive and social maturity (Menabò & Guarini, 2025), while media and social narratives reinforce a simplified bully–victim dichotomy (Gini et al., 2021; Scott et al., 2025). Consequently, the relational and systemic dimension of cyberbullying risks remaining marginal in their representations. Within this framework, references to pro-bullies, which emerged as a typical category in our data, take on particular relevance. Students recognized that supporting the bully may function as a strategy of self-protection: joining the aggressor to avoid becoming the next victim. This acknowledgment suggests that adolescents implicitly understand the logic of shared and negotiated power, in which alignment with the cyberbully becomes an investment in security, a dynamic also observed in traditional bullying (Milnes et al., 2022).

At the same time, although to a lesser extent, participants referred to passive bystanders, criticizing them for their inaction and accusing them of failing to support the victim. This critical stance reveals a form of normative awareness: adolescents recognize the potential transformative power of those who “do nothing.” Yet, this critique was not accompanied by positive recognition of the defender role, which was entirely absent from their narratives. This absence may be attributed to at least two factors. First, there is an asymmetry in role visibility: passive bystanders constitute the majority and appear in almost every bullying episode, whereas defenders are comparatively rare and often intervene in less visible ways—for instance, by privately comforting the victim (Lambe & Craig, 2020; Zhao et al., 2023). Second, a gray area of responsibility exists: although the moral duty to “do something” is generally recognized, the lack of social models and concrete experiences makes the defender role less feasible and socially legitimized (Menabò & Guarini, 2025; Thornberg et al., 2022).

### 4.4. Coping Strategies

Regarding bystanders’ coping strategies, adolescents reported that defending victims in digital contexts is perceived as a challenging task, a finding that aligns with previous literature. Participants highlighted anonymity and the difficulty of identifying the perpetrator as key obstacles to intervention. Some stated they would only act if they personally knew the victim, while others expressed hesitation due to uncertainty about who was behind the harassment.

Regarding victim coping strategies, adolescents in our study described a broad range of responses that mirror established typologies in the literature. Technical strategies such as deleting or hiding offensive content and blocking the aggressor were frequently adopted to protect one’s online identity and reduce contact. Confrontational responses were also reported, though most participants stressed the importance of reacting without exacerbating the conflict. Many adolescents referred to social strategies, including confiding in trusted adults or friends, confirming previous evidence on the relevance of interpersonal support (Biernesser et al., 2023; Jacobs et al., 2015). At the same time, participants expressed reluctance to involve their parents, citing fears of losing access to social media or not being understood, concerns that echo previous research (Martin et al., 2023).

Avoidant or passive strategies, such as ignoring the abuse or taking a break from social media, were often used to preserve emotional well-being, in line with the literature on functional withdrawal as a form of protection (Midamba & Moreno, 2018; Smith et al., 2008).

Beyond these points of continuity, our findings also extend and enrich current theoretical frameworks. A notable contribution is the clear differentiation between bystander and victim strategies: while much of the literature focuses on the victim’s perspective, our data show how bystanders experience moral conflict and inhibition, often due to perceived helplessness and lack of connection with the victim. Adolescents also displayed a nuanced perception of strategy effectiveness, critically evaluating actions such as blocking, which some feared could provoke the bully, and noting the limitations of posting defensive comments, which could be deleted instantly. These reflections reveal a more tactical and context-aware approach to coping, rarely explored in depth in prior studies.

Another innovative finding concerns the role of online reputation management. Several adolescents described coping strategies such as deleting photos not only to reduce harm but also to protect their public image, highlighting how digital self-presentation influences coping decisions. Additionally, some adolescents employed subtle, non-aggressive forms of confrontation, such as ironic or sarcastic replies, demonstrating a developmentally attuned strategy of self-defense that avoids direct escalation, an aspect not widely addressed in existing models.

Help-seeking behaviors also emerged as conditional and selective. While many participants acknowledged the value of support from adults or friends, they often weighed this against potential negative outcomes, such as restrictions on phone use or being misunderstood. This reveals a complex cost–benefit assessment that guides whether or not adolescents reach out for help. Finally, our results underscore the flexible and adaptive nature of coping. Adolescents showed the ability to shift across strategies, reporting, blocking, ignoring, confronting, depending on the context, which reflects the dynamic model of coping described by Raskauskas and Huynh (2015).

In conclusion, coping with cyberbullying among adolescents appears to involve a deliberate balance between self-protection, reputation management, social support, and conflict regulation, all within a digital environment shaped by anonymity, permanence, and the immediacy of content.

### 4.5. Limitations

While this study provides valuable insights into adolescents’ perceptions and experiences of cyberbullying, several limitations should be acknowledged. First, the study relied on focus groups conducted within school settings, where the relatively large number of participants may have limited the opportunity for all students to express themselves fully. Although this approach allowed for rich group discussions and spontaneous exchanges among peers, it may have constrained the expression of highly personal or sensitive experiences, especially given the stigmatized and often private nature of online victimization. Some participants may have hesitated to disclose episodes involving classmates or to discuss potentially distressing content in front of others. This limitation is consistent with previous research indicating that discussions of bullying may inhibit the disclosure of sensitive experiences (Guerra et al., 2011; Vessey et al., 2017). Future research could combine focus groups with individual interviews or anonymous digital diaries to capture more personal reflections and to ensure that more private experiences are represented.

Second, as discussions focused on spontaneous definitions, behaviors, and coping strategies, the breadth of the explored themes may have come at the expense of depth in certain areas. For instance, some aspects, such as the emotional regulation processes underlying coping strategies, and the interplay between online and offline forms of aggression, were only indirectly addressed. This difficult balance between the range of topics discussed and the depth of exploration is a well-recognized limitation in qualitative research, particularly in focus group designs (Krueger & Casey, 2015). Therefore, future studies could explore these dimensions through more targeted qualitative designs or mixed-method approaches, integrating interviews with observational or digital trace data.

Third, while the study provides an updated snapshot of adolescents’ views, the constantly evolving nature of digital platforms means that the forms and contexts of cyberbullying can change rapidly, as also observed in research concerning cyberbullying (Phanniphong et al., 2024; Scheithauer et al., 2021). The platforms most frequently mentioned by students, such as TikTok, Instagram, and WhatsApp, reflect the social media landscape at the time of data collection, but newer platforms or features (e.g., anonymous messaging, AI-generated content) may shape future expressions of cyberbullying differently.

Finally, as the study focused primarily on students’ perspectives, it did not include the viewpoints of other key stakeholders such as parents, teachers, or school staff, despite previous research highlighting the importance of incorporating these perspectives to achieve a more comprehensive understanding of cyberbullying (e.g., Khairi et al., 2022; Niven et al., 2025). Incorporating these viewpoints would enable a more systemic understanding of cyberbullying as a multi-layered phenomenon, encompassing not only individual perceptions but also institutional responses, adult mediation practices, and policy frameworks.

### 4.6. Implications

This study highlights several key implications for advancing the understanding of how adolescents define cyberbullying, describe behaviors, recognize roles, and navigate coping strategies. At the definitional level, cyberbullying is seen as a form of hostile communication, but its impact becomes particularly intense when the content is disseminated publicly, comes from someone close, or carries a clear intention to humiliate the victim. Likewise, although cyberbullying was originally framed within the traditional definition of bullying, new dimensions specific to the digital context, such as publicity and anonymity, have progressively emerged. Yet, these factors do not appear to hold the same relevance for students: while anonymity may intensify feelings of vulnerability, the publicity of harmful content clearly dominates adolescents’ accounts as the element that most amplifies the impact of online aggression. Exploring these nuances will allow academic definitions to be better aligned with how young people actually experience this phenomenon. Furthermore, the results indicate that cyberbullying behaviors cannot be analyzed in isolation from the digital environment in which they occur. The platform and its implicit social norms influence the perceived severity and emotional experience of the behavior. This opens a line of research focused on the interaction between the type of behavior, digital medium, and relational context. Exploring how certain actions acquire different meanings depending on the channel, for example, private messages on WhatsApp versus posts on public platforms like TikTok or Instagram, would allow a more precise and situated understanding of the phenomenon, with clear implications for prevention and risk assessment. At the same time, since adolescents actively use multiple digital channels, future prevention and educational initiatives should integrate the platforms they most frequently engage with (e.g., TikTok, Instagram, WhatsApp), not only as contexts of risk but also as potential tools for positive communication. Promoting a critical and responsible use of these media can help young people recognize harmful behaviors and actively contribute to creating supportive online environments. In addition, the rapid evolution of digital technologies and the emergence of artificial intelligence tools capable of generating, modifying, or disseminating content pose new challenges for the study of cyberbullying. Keeping research and intervention programs up to date with these technological developments is essential to identify new forms of aggression and misinformation, which may amplify harm or obscure responsibility.

Since adolescents tend to understand it mainly as a direct confrontation between aggressor and victim, it is essential to broaden their perspective to include other key actors, such as pro-bullies. passive bystanders, and especially active defenders. Fostering this awareness can empower young people to take a more active role in preventing and mitigating cyberbullying, especially by encouraging the defender’s role as an agent of change within their digital communities. Finally, another fundamental intervention line is the development of specific programs that not only inform adolescents about possible coping strategies for cyberbullying but also train them in their practical application. Additionally, it is crucial to incorporate emotional support spaces and strengthen trust networks with significant adults, such as parents, teachers, and school counselors, to overcome the fear or mistrust that often prevents action. Our results show that although young people know possible ways to act, they often do not apply them due to insecurity or lack of confidence. Therefore, these programs must close the gap between knowledge and action by teaching how to assess specific contexts and choose the most appropriate response, promoting adaptive and effective management of bullying in digital environments.

Finally, future research should further investigate how platform characteristics, digital social capital (e.g., visibility and follower dynamics), and emerging technologies such as artificial intelligence shape both vulnerability and intervention behaviors across diverse contexts.

## 5. Conclusions

This study deepened the understanding of how adolescents define, identify behaviours and roles, and cope with cyberbullying by giving them a direct voice, through a consensual qualitative approach. Concerning the first research question on how adolescents define and conceptualise cyberbullying, our study suggests that students depicted cyberbullying as a multifaceted phenomenon marked by aggression, anonymity, and the uncontrolled spread of harmful content. Notably, the prominence attributed to the public dissemination of aggressive content suggests that academic definitions may need to more explicitly incorporate the relational and reputational impact of visibility in digital environments. In addressing the second research question regarding which behaviors adolescents identify as cyberbullying, participants primarily referred to direct and visible actions, such as insults, threats, and the non-consensual sharing of images, revealing a focus on overt manifestations of online aggression. These findings indicate that prevention efforts should move beyond generic awareness campaigns and instead address platform-specific norms and the reputational dynamics that amplify harm across different digital contexts. In relation to the third research question concerning how adolescents perceive and describe the roles involved in cyberbullying situations, participants’ narratives revealed a predominantly dyadic view focused on the cyberbully and the cybervictim, with limited recognition of other roles, such as defenders. This suggests the importance of broadening adolescents’ understanding of the group-based nature of cyberbullying by explicitly fostering awareness of pro-bullies, passive bystanders, and especially active defenders as potential agents of change within digital communities. With regard to the final research question on the coping strategies reported by adolescents, the findings revealed a range of varied and adaptive responses, reflecting both awareness and hesitation in taking action, particularly among bystanders. The conditional and strategic nature of adolescents’ coping responses highlights the need for school-based programs that train them in context-sensitive decision-making, reputation management, and effective intervention skills. Strengthening trust networks with teachers, parents, and school counsellors is essential to reducing fears of misunderstanding or punitive consequences that often inhibit help-seeking.

In conclusion, although adolescents demonstrate a nuanced understanding of cyberbullying, their perspectives remain partial, highlighting the need for educational initiatives that foster empathy, digital responsibility, and effective intervention skills. These findings call for refined conceptual definitions that explicitly address visibility and reputational impact, and a platform-sensitive and technologically updated approach. Strengthening defender awareness and context-sensitive coping strategies within school-based programs is also essential. By centering students’ voices, this study advances a more grounded and relationally informed understanding of cyberbullying in contemporary digital contexts.

## Figures and Tables

**Table 1:** Domains and Categories.

Domain	Category		
	General	Typical	Variant
Definition of Cyberbullying	“Hostile online communication” “Uncontrolled spread”	“Anonymity and concealment”	“No need for repetition”
Cyberbullying Behaviors	“Variety of platforms” “Unpleasant comments and insults” “Threats” “Sharing embarrassing content”	“ID theft/impersonation” “Fake identity”	“Dangerous challenges” “Severity differences”
Roles Involved	“Cyberbully’s profile” “Cybervictim’s profile”	“Pro-bullies”	“Passive Bystanders”
Coping Strategies	“Victim strategies”	“Bystander strategies”	

Note. This table presents the domains that emerged along with the categories identified for each domain. Each category is categorized as typical, general, or variant. No variant category was identified for the domain “Coping Strategies”.

## Data Availability

The data collected in the research (interview transcripts in Italian) are available to other researchers upon reasonable request.
